# A Comparative Study on the Carcass and Meat Chemical Composition, and Lipid-Metabolism-Related Gene Expression in Korean Hanwoo and Brindle Chikso Cattle

**DOI:** 10.3390/cimb45040214

**Published:** 2023-04-07

**Authors:** Van-Ba Hoa, Dong-Heon Song, Kuk-Hwan Seol, Sun-Moon Kang, Hyun-Wook Kim, In-Seon Bae, Eun-Sung Kim, Yeon-Soo Park, Soo-Hyun Cho

**Affiliations:** 1 Animal Products Utilization Division, National Institute of Animal Science, RDA, Wanju 55365, Republic of Korea; 2 Jeonbuk Livestock Research Center, Jinan-Gun 55460, Republic of Korea; 3 Gangwon-do Livestock Research Institute, Hoengseong-Gun 25266, Republic of Korea

**Keywords:** cattle breed, marbling, intramuscular fat, fatty acid, gene expression

## Abstract

The objective of this study was to elucidate the effect of cattle breed on carcass and meat chemical composition, fatty acid profiles, and lipid-metabolism-related genes. For this study, same-age Hanwoo and Chikso steers (*n* = 6 per breed) reared under identical conditions were used. Immediately after slaughter, muscle tissues were collected for analysis of mRNA expression. At 24 h post-mortem, the carcasses were assessed for carcass traits (marbling score, meat yield, etc.), and meat quality and fatty acid profiles in the *longissimus lumborum* (LL) and *semimembranosus* (SM) muscles. The results showed that no differences in the slaughter weight, dressing rate, back-fat thickness, trimmed fat, and total meat yield occurred between the two breeds (*p* > 0.05). However, Hanwoo cattle had a higher marbling score, intramuscular fat (IMF) content, and expression level of lipid-metabolism-related genes such as lipoprotein lipase, peroxisome proliferator-activated receptor gamma, and fatty acid binding protein 4, compared with Chikso (*p* < 0.05). Contrastingly, Chikso had a higher total unsaturated fatty acid content and expression level of stearoyl CoA desaturase 1 (*p* < 0.05). It may be said that the difference in the expression levels of lipid-metabolism-related genes could be the molecular factors underlying IMF deposition and fatty acid profile differences in the beef from the two breeds.

## 1. Introduction

In many markets, intramuscular fat (IMF) or marbling degree (flecks of IMF in ribeye muscles) is considered as the most important determinant of purchasing decision by consumers for beef [1,2,3], because IMF content positively affects the eating qualities of beef, such as tenderness, juiciness, and flavor [4,5,6]. Additionally, the fatty acid composition of beef has received considerable interest due to its effects on human health and the organoleptic characteristics of the beef [7,8,9]. Researchers have reported that the IMF and composition of fatty acids in beef are impacted by extrinsic factors such as genetics and feeding conditions [10,11]. It is known that adipogenesis and de novo fatty acid synthesis are the major pathways of fat deposition [5], which are tightly regulated by adipogenic transcription factors, such as peroxisome proliferator-activated receptor gamma (PPAR), fatty acid binding protein 4 (FABP4), stearoyl-CoA desaturase 1 (SCD1), acetyl-CoA carboxylase (ACACA), etc. [5,12,13].

Hanwoo is the major beef cattle in Korea [1]. It has been reported to some foreign markets, such as Hong Kong since 2015, because of its high IMF level and the unique palatability of its meat [1,14]. Besides the Hanwoo, another cattle breed named Chikso (Korean native brindle cattle) registered with the Domestic Animal Diversity Information System of the Food and Agriculture Organization, has recently been used as beef cattle in Korea [15,16]. Chikso is characterized by its unique brindle coat color [17], which is completely different from the other registered cattle breeds. Compared to the popular commercial Hanwoo raising in Korea, however, the Chikso breed is generally maintained at a smaller population size, with around 4000 heads in 2016 [18]. In recent years, Chikso has been recognized as a valuable breed and has received more attention from beef producers due to an increasing demand for safe meat products derived from native cattle breeds in South Korea [19]. However, to promote the production and commercialization of Chikso beef, a lot of assessments, such as the effects of genetics, environment, nutrition, etc., on the growth performance, carcass traits, meat yield, marbling degree, and meat quality (e.g., IMF), are needed because all of these factors directly affect the economic efficiency of beef production and consumer demand [1]. To the best of our knowledge, only a few studies have been conducted to characterize the genetic diversity of Chikso [16,17], and little scientific information regarding the carcass traits, meat yield, IMF, and fatty acid profiles in Chikso beef is available.

Beef breeds may have different capabilities for adipose tissue accumulation [10,11]. However, it still remains known whether there is a difference between the Hanwoo and Chikso in IMF content, fatty acid profiles, and molecular factors underlying lipid metabolism. Therefore, the aim of this study was to elucidate the effect of cattle breed (Chikso and Hanwoo) on carcass traits, meat chemical composition, and lipid-metabolism-related genes.

## 2. Materials and Methods

### 2.1. Animals

A total of 12 cattle (6 Hanwoo and 6 Chikso) purchased from a commercial cattle farm (Kangwon, Korea) were used in the present investigation. The information regarding the animals (age, feeding regime, feed, etc.) was collected from the farm household as follows: All the animals were surgically castrated at 7 months old, and housed in two different pens (ca. 10 m^2^ per animal) in the feedlot of the farm. The steers were reared under identical conditions: during the growing phase the animals were fed twice/day with available commercial feed (3.0–7.5 kg) and rice straw (3.0–4.0 kg). During the fattening phase (18 months of age to slaughter), the animals were fed ad libitum with a high rate (90%) of the commercial feed (72–73% total digestible nutrients and 11–12% crude protein) and only 10% rice straw. The steers were collected at 30 months of age and were shipped to an abattoir of the National Institute of Animal Science (NIAS, Wanju-gun, Korea) for a duration of 2 h, where they were fasted for about 12 h. The animals were slaughtered using an industry-accepted procedure. Immediately after bleeding and skinning, approximately 20 g of muscle tissue (*longissimus lumborum* at the 13th lumbar vertebra) were taken from the left side of each animal, frozen in liquid nitrogen, and subsequently stored at −80 °C for determination of lipid-metabolism-related gene expression. All procedures were approved by the Institutional Animal Care and Use Committee (IACUC) of NIAS (Approval No. NIAS 20001992). After slaughter, the carcasses were chilled at 2 °C in a chilling room.

### 2.2. Carcass Traits and Meat Yield

At 24 h post-mortem, the weight of each carcass was recorded to determine the dressing rate (by dividing the cold carcass weight by live weight multiplied 100). Thereafter, a transversal cut on the *longissimus dorsi* muscle between the 12th and 13th ribs was carried out on the left side of each carcass to evaluate back-fat thickness using a Vernier caliper, ribeye area using a ribeye grid, and beef marbling score (BMS: values of 1 and 9 indicate the poorest and greatest degree of marbling, respectively) by an official grader. Finally, both the left and right sides of carcasses were brought to the cutting room where they were fabricated into primal and sub-primal cuts following the Korean Hanwoo Beef Specification Guide [20]. The cuts were deboned and trimmed of visual fat (subcutaneous and intermuscular fat) and connective tissues. Thereafter, the meat content of each sub-primal cut was weighed to determine its yield (meat weight divided by cold carcass weight multiplied by 100). The total meat yield per carcass was expressed in weight (total meat weight from all sub-primal cuts, kg) and as a percentage (total meat weight divided by cold carcass weight multiplied by 100, %). Moreover, the trimmed fat and bones from all cuts were collected and weighed to determine their yield per carcass. After the meat yield determination was completed, the *longissimus lumborum* (LL) and *semimembranosus* (SM) from the left side of Hanwoo and Chikso carcasses were collected for chemical composition and fatty acid analysis. After the trimming of all visual fat and connective tissues, each muscle was cut into sub-samples depending on each analysis.

### 2.3. Chemical Composition

The chemical composition (protein, crude fat, moisture, collagen, and ash) of the meat samples was determined using a Food Scan™ Lab 78810 (Foss Tecator Co., Ltd., Hillerød, Denmark), as described by Anderson [21]. Before analysis, the meat samples were trimmed of all visual subcutaneous fat and outer connective tissues, and chopped and ground using a grinder (Hanil Co., Dangjin-si, Chungcheongnam-do, Korea). Next, triplicate aliquots (about 200 g) of each sample were taken and distributed onto a round dish which was then loaded into the sample chamber of the device. The contents of protein, fat, moisture, collagen, and ash were expressed as percentages (%) of the total.

### 2.4. Fatty Acid Composition

The lipid content in the meat samples was extracted following the method described in our previous study [22]. Thereafter, the methyl esters from fatty acids (FAMES) were formed using a solution mixture containing 1 mL tricosanoic acid and 1 mL of 0.5 N NaOH. The FAMES were analyzed using a CP-3800 gas chromatograph/flame ionization detector (GC-FID, Bruker, Bremen, German) equipped with an Omegawax™ 320 fused capillary column (30 m × 0.25 mm × 0.25 µm film thickness; Supelco, Bellefonte, PA, USA). The GC conditions set were: initial oven temperature at 50 °C for 1 min, increased at a rate of 25 °C/min to 200 °C, and finally raised at a rate of 5 °C/min to 230 °C. The injection port and detector temperatures set were 250 and 260 °C, respectively. Fatty acids were identified by comparing their relative retention times with those of fatty acid methyl ester standards (Sigma, St. Louis, MO, USA). Individual fatty acids were expressed as a relative percentage (%) of total fatty acids. Each sample was analyzed in duplicate.

### 2.5. Adipogenesis-Related Genes Expression

To assess whether there is a difference in the expression of the adipogenesis-related genes between the two cattle breeds, immediately after bleeding and skinning the LL muscle tissues were collected and used for mRNA expression. Total RNA in the LL muscle tissue was extracted using TRIzol reagent (Sigma-Aldrich, Carlsbad, CA, USA) according to the manufacturer’s instructions. Thereafter, 1 μg of total RNA from each sample was reversely transcribed into cDNA using an iScript™ cDNA Synthesis Kit (Bio-Rad, Seoul, Republic of Korea). Real-time polymerase chain reaction (RT-PCR) was carried out using SSoAdvanced Universal SYBR Green Supermix (Bio-Rad, Seoul, Republic of Korea), a cDNA equivalent of 10 ng of total RNA from each sample, and primers specific to bovine lipoprotein lipase (LPL), peroxisome proliferator-activated receptor gamma (PPARG), acetyl-CoA-carboxylase alpha (ACACA), stearoyl CoA desaturase 1 (SCD1), fatty acid binding protein 4 (FABP4), and glyceraldehyde-3-phosphate dehydrogenase (GAPDH) (Table 1). The primers were designed using a free online designing tool (http://www.ncbi.nlm.nih.gov/tools/primer-blast/index.cgi, accessed on 20 November 2022). The thermal conditions for the amplification of genes were initial annealing at 57 °C for 2 min, denaturation at 95 °C for 3 min, followed by 40 cycles of denaturation and annealing at 95 °C/10 s and 60 °C/10 s, respectively. The data were normalized by the GAPDH internal control, and the relative expression of each gene was calculated using the 2^−ΔCt^ method [23].

### 2.6. Statistical Analysis

Statistical analysis was conducted using SAS Enterprise software (version 7.1; SAS Inst. Inc., Cary, NY, USA). The data were analyzed using the general linear model procedure where the cattle breed was considered as a fixed effect, and the carcass traits (slaughter weight, hot carcass weight, cold carcass weight, dressing rate, back-fat thickness, marbling score, and ribeye area), meat yields of sub-primal cuts, chemical composition (protein, moisture, intramuscular fat, collagen, and ash), fatty acids, and mRNA expressions of genes were considered as dependent variables. The chemical composition and fatty acid profiles of all the meat samples were analyzed in duplicates. The mean difference was compared using Duncan’s multiple range test and significant differences were set at a 5% level. The data are presented as means ± standard deviation.

## 3. Results and Discussion

### 3.1. Carcass Traits and Composition

In Korea, the major sex type of cattle beef is steer that is usually slaughtered at around 26 to 30 months of age [24]. In the present study, both Chikso and Hanwoo steers were harvested at the same age (30 months) and their carcass traits and composition are presented in Table 2. The results showed that no differences occurred in slaughter weight, hot and cold carcass weight, and dressing rate between the two cattle types (*p* > 0.05). The carcass weights in both cattle types fell in the average range (around 430 kg) reported for Hanwoo steers harvested at around 30 months of age by the Korea Institute of Animal Products Quality Evaluation (KAPE) [25]. These results imply that both cattle breeds showed a similar growth potential under identical rearing conditions. Regarding the carcass composition, the trimmed fat and bone yields did not differ between the cattle types (*p* > 0.05). Compared with our data, those of Gotoh et al. [26] found a higher trimmed fat (208 kg) and lower bone weight (53 kg) in Japanese black steers harvested at 744 kg body weight.

Notably, the marbling showed a significant difference between the breeds. According to the Korean beef marbling score (BMS) ranging from 1 (devoid) to 9 (abundant) [25], the Hanwoo steers had a higher BMS score (6.33, corresponding to a moderate degree) compared to the Chikso (2.83, corresponding to a trace marbling degree) (*p* < 0.05). In many countries (e.g., Korea, Japan, USA, etc.), marbling is considered the major determinant of carcass quality grade and market price because it is an indicator of beef eating quality [27,28,29]. According to the Korean Beef Grading Standard of the Korean Institute for Animal Products Quality Evaluation [25], the BMS of Hanwoo carcasses obtained in this study fell within the 1^++^ quality grade while those of Chikso belonged to the quality grade 2 [6,27]. The reason for the difference in results between the two cattle breeds could be related to the genetic differences d which the Hanwoo seemed to have a higher potential for marbling accumulation compared to the Chikso [6]. In animals, fat deposition occurs in some depots including subcutaneous, abdominal cavity, intermuscular and intramuscular locations. The rate and proportion of fat deposition in the depots differ depending on the animal age, species and breed, and feeding regime [11,26,30]. Based on these results, therefore, it may be said that the patterns of subcutaneous and intermuscular fat accretion were similar in both the cattle types studied but the intramuscular fat deposition was remarkably greater in the Hanwoo compared to the Chikso.

### 3.2. Meat Yield

In Korea and other countries, a beef carcass is usually fabricated into various cuts, depending on consumer preferences [31]. The meat yields in terms of weight (kg) and percentage (%) of sub-primal cuts from Chikso and Hanwoo are presented in Table 3. In general, it was observed that in almost all cuts the meat yield was not different between the two breeds. Some cuts which showed significant differences in the meat yields between the breeds were: lower loin (10th–13th vertebra), chuck flap striploin, the eye of round, outside round head, knuckle, short blade, and inside skirt (*p* < 0.05). Among these, the brisket point end had the highest meat yield, followed by chuck roll, top round, upper loin, and the heel meat center had the lowest. The total meat yield was 237.50 and 247.44 kg, which corresponds to 57.31 and 53.99% in the Chikso and Hanwoo, respectively, and no noteworthy dissimilarity was found between the two breeds (*p* > 0.05). Compared to our results, those of Gotoh et al. [26] found a slightly lower meat yield (238 kg) in Japanese black steers finished on a concentrate-based diet and slaughtered at a similar body weight (744 kg). Clinquart et al. [32] showed that gender, age, and feeding system are the major factors affecting beef cut yield. In our study, we kept the feeding regime, age, and gender constant for both breeds, and therefore the results indicating no differences in the meat yield could be related to the similar genetic potential for muscle accretion in both cattle breeds.

### 3.3. Proximate Composition

The determination of proximate chemical composition (e.g., fat, moisture, protein, collagen, and ash) of meat allows the identification of factors associated with genetics, age, feeding diet, etc. of animals before slaughter, and it closely relates to almost all quality traits of the meat, such as water holding capacity, color, tenderness, juiciness, and flavor [24,33]. In the present study, the proximate composition was determined on both muscle types (LL and SM) from the cattle breeds, and the results are presented in Table 4. In both muscles, significant differences were found in protein, moisture, fat, collagen, and ash between the two cattle breeds (*p* < 0.05). Particularly, the Hanwoo beef contained higher IMF (by approximately three times in the LL muscle) and lower moisture content compared to the Chikso beef (*p* < 0.05). The IMF level (23.96%) in the LL muscles of Hanwoo in this study fell within the fat range (over 20%) for the highly-marbled Hanwoo beef class [33,34]. Compared with our data, the study by Okumura et al. [35] found a similar fat level (23.3%) in the *longissimus* muscles of highly-accumulated fat Japanese black steers fed a concentrate diet. However, the fat levels in both muscles from the Chikso in the present study were still higher compared to those reported for *longissimus* muscles from the Belgian Blue, German Angus, and Holstein–Friesian breeds [26].

**Figure 1:**
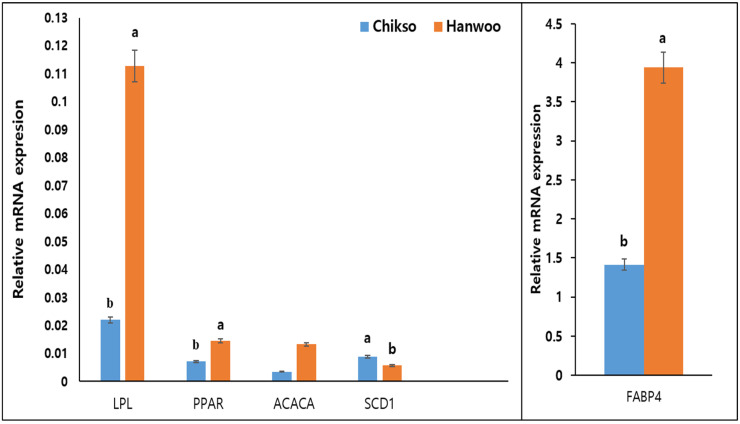
The effect of cattle breed on the expression of lipid-metabolism-related genes: lipoprotein lipase (LPL), peroxisome proliferator-activated receptor gamma (PPARG), acetyl-CoA-carboxylase alpha (ACACA), stearoyl CoA desaturase 1 (SCD1), and fatty acid binding protein 4 (FABP4) in the LL muscles. The expression levels were calculated using the 2^−ΔCt^ method and were normalized to the glyceraldehyde-3-phosphate dehydrogenase (GAPDH). Different letters (a, b) indicate the statistical difference (*p* < 0.05) between the two cattle breeds.

It has long been recognized that the IMF content plays a crucial role in the eating quality of beef since it is positively correlated to almost all the eating quality traits, such as tenderness, flavor, and juiciness [24,27,33]. Compared to Chikso, the higher IMF level in Hanwoo beef could be the consequence of a long-term strategic genetic improvement program [36]. Hitherto, relationships have been reported between the adipogenesis process or fat accumulation in beef and the regulation of related genes [37], and peroxisome proliferator-activated receptor gamma (PPAR*g*), fatty-acid-binding protein 4 (FABP4), and lipoprotein lipase (LPL) play the most important role in this process [12,38,39]. The fat or adipose tissues in animal carcasses are deposited in different areas such as subcutaneous, intermuscular, and intramuscular depots [40]. As expected, the mRNA expressions of PPARg, FABP4, and LPL were significantly higher in the Hanwoo compared to those in Chikso (Figure 1) (*p* < 0.05). These results were in line with the IMF content in the LL and SM muscles (Table 4). The PPAR*g* regulates the adipogenesis pathway by promoting the differentiation of pre-adipocytes into mature adipocytes [41]. The FABP4, exclusively expressed in adipocytes within the genomic regions of quantitative trait loci, is considered the genetic marker for marbling and IMF deposition in many cattle breeds (e.g., Korean and Australian cattle) [42]. On the other hand, LPL is an important enzyme that hydrolyzes the adipogenesis-derived triglycerides into fatty acids which are subsequently deposited into adipose tissues [43]. Consistent with our results, other studies have also reported that beef cattle breeds (e.g., Japanese black cattle) with higher IMF contents are associated with a greater expression of PPARg and FABP4 compared to low accumulated fat cattle breeds [38]. Overall, it may be said that the differences found in the IMF and marbling degree could be attributed to the genetic differences in terms of which the Hanwoo cattle exhibited a greater potential for IMF accumulation compared to the Chikso.

### 3.4. Fatty Acid Profiles

The fatty acid composition of LL and SM muscles from two cattle types is presented in Table 5. Out of thirteen identified fatty acids, palmitic acid (C16:0), cis-vaccenic acid (C18:1n7), linoleic acid (C18:2n6), and gamma linoleic acid (C18:3n6) were significantly (*p* < 0.05) affected by the cattle breed. Among the saturated fatty acids (SFA), palmitic acid (C16:0) and stearic acid (C18:0) were the most predominant fatty acids in the beef from both cattle breeds. However, Hanwoo beef had a significantly higher C16:0 level (31.18 and 32.51% in the LL and SM muscles, respectively) compared to that of Chikso beef (27.22 and 28.18% in the LL and SM muscles, respectively) (*p* < 0.05). In livestock in general and ruminants in particular, palmitic acid is synthesized from substrates such as malonyl-CoA and acetyl-CoA under the regulation of acetyl-CoA carboxylase alpha (ACACA) in the de novo fatty acid synthesis pathway [12,37]. The expression of the ACACA gene was slightly greater in the Hanwoo than in the Chikso but not significantly different (*p* > 0.05) (Figure 1). As a result of the greater C16:0 level, the total SFA content was also higher in both the muscles of Hanwoo, compared with Chikso beef (*p* < 0.05). Oleic acid (C18:1n9) was the most predominant monounsaturated fatty acid (MUFA) in both muscles from the two cattle breeds. However, the difference was only found in the SM muscle, meaning that the synthesis of these fatty acids was affected not only by the breed but also the anatomical location (muscle type). Oleic acid has long been recognized as the important flavor precursor which produces a variety of volatile compounds contributing to desirable aromas (fatty odor notes) of cooked meat during cooking [8]. Besides the MUFAs, the level of individual polyunsaturated fatty acids (PUFA) such as C18:2n6 and C18:3n6 as well as total PUFA content were significantly higher in the Chikso, compared with the Hanwoo beef (*p* < 0.05). The unsaturated fatty acids (UFA) content was higher in both muscles from the Chikso (*p* < 0.05). It is known that the UFAs are synthesized in the de novo fatty acid synthesis pathway that is regulated by stearoyl-CoA desaturase-1 (SCD1) or by an action of different enzymes encoded by a number of genes [37]. Particularly, SCD1 regulates the conversion of SFAs into UFAs in this pathway. It was observed that the mRNA expression of SCD1 was higher in the Chikso compared to the Hanwoo beef (*p* < 0.05). According to previous studies [44,45], the *longissimus thoracis* muscles (16–20% fat) of Hanwoo steers slaughtered at 30–32 months of age had similar oleic acid (47%), SFA (46%), UFA (53%), and MUFA (50–52%) contents compared with those in the Hanwoo cattle in the present study. The fatty acid profiles of meat reflect not only its nutritional value but also influence the flavor characteristics of cooked meat during cooking. Particularly, during cooking, the MUFAs (e.g., oleic acid) undergo the thermal oxidation process which produces a variety of volatile compounds contributing to cooked meat flavors [8]. According to the recommendations of the World Health Organization for a healthy diet, a reduced intake of SFAs and an increased intake of PUFAs are strongly encouraged, the PUFA/SFA ratio should be 0.40 or higher, and the n6/n3 fatty acid ratio should be 4.0 or lower [46]. Based on the outcome of the fatty acid analysis, both the Chikso and Hanwoo beef had a PUFA/SFA ratio that was lower than the recommended value, and the Chikso beef (in the case of the LL muscle) showed a higher PUFA/SFA ratio. However, compared with our data, the study by Coleman et al. [47] found higher SFA (47–49%) and lower MUFA (45–47%) contents as well as a lower PUFA/SFA ratio (0.1–0.12) in LL muscles from other cattle breeds.

## 4. Conclusions

This study assessed the effect of cattle breed on the carcass characteristics, chemical composition, fatty acid profile, and expression of lipid-metabolism-related genes in beef raised under identical conditions. The breed did not affect the total trimmed fat and meat yield, but it did affect the marbling score and intramuscular fat content as well as the fatty acid profile in meat. Additionally, the breed also affected the expression pattern of lipid-metabolism-related genes such as PPAR, LPL, and FABP4. Based on the results obtained from the study, it may be said that there was a difference between the two cattle breeds in the genetic potential for adipose tissue accumulation which affected the IMF levels and fatty acid profiles in the beef. Furthermore, the PPAR, LPL, and FABP4 could be considered the key molecular factors underlying the fat deposition and fatty acid synthesis in beef. Further study is needed to elucidate the effect of breed on muscle fiber types, nutritional characteristics, taste, and aroma markers in beef.

## Figures and Tables

**Table 1:** Primer sequences used for the amplification of lipid-metabolism-related genes in this study.

Gene Name	Primer Sequence (5′–3′) *	Accession No.	Product Size (bp)
Lipoprotein lipase (LPL)	Forward: AGCTCCAAGTCGCCTTTCTC	BC118091	181
	Reverse: TGCAATCACACGGAGAGCTT		
Peroxisome proliferator activated receptor gamma (PPARG)	Forward: ACTTTGGGATCAGCTCCGTG	NM_181024	115
	Reverse: TCCTCATAGTGCGGAGTGGA		
Acetyl-CoA-carboxylase alpha (ACACA)	Forward: ACGGCTGACTGGAGTTGAAG	AJ132890	336
	Reverse: AATCGGGAGTGCTGGTTCAG		
Stearoyl CoA desaturase 1 (SCD1)	Forward: TCACATTGATCCCCACCTGC	AF188710	962
	Reverse: TCACATTGATCCCCACCTGC		
Fatty acid binding protein 4 (FABP4)	Forward: GCATGGCCAAACCCACTTTG	NM_174314	107
	Reverse: TTCCTGGCCCAATTTGAAGGA		
Glyceraldehyde-3-phosphate dehydrogenase (GAPDH)	Forward: GGTCACCAGGGCTGCTTTTA	NM_001034034	222
	Reverse: CCAGCATCACCCCACTTGAT		

* The primer sequences used were designed using a free online designing tool (http://www.ncbi.nlm.nih.gov/tools/primer-blast/index.cgi (accessed on 20 November 2022)).

**Table 2:** The effect of cattle breed on carcass characteristics.

Traits	Chikso (*n* = 6)	Hanwoo (*n* = 6)
Slaughter weight (kg)	679.92 ± 76.04	745.83 ± 78.70
Hot carcass weight (kg)	423.58 ± 50.28	466.29 ± 60.31
Cold carcass weight (kg)	417.30 ± 50.02	459.83 ± 60.30
Dressing rate (%)	62.30 ± 5.12	62.60 ± 5.61
Back-fat thickness (mm) at 12th rib	14.50 ± 6.66	12.00 ± 4.73
Marbling score (1–9) *	2.83 ± 1.83 ^b^	6.33 ± 2.07 ^a^
Ribeye area (cm^2^)	87.00 ± 9.44	88.83 ± 4.22
Trimmed fat (kg)	120.59 ± 32.80	137.40 ± 31.07
Trimmed fat (%)	28.53 ± 5.21	29.61 ± 3.21
Bone (kg)	67.29 ± 6.60	73.05 ± 6.42
Bone (%)	16.27 ± 2.21	15.97 ± 0.92

Means within a row with different superscripts (a, b) differ significantly (*p* < 0.05). * Marbling score: 1—devoid; 9—abundant.

**Table 3:** The effect of cattle breed on the meat yield of sub-primal cuts.

Sub-Primal Cuts	Meat Yield in Weight (kg)		Meat Yield in Percentage (%)	
	Chikso	Hanwoo	Chikso	Hanwoo
Tenderloin	7.05 ± 0.48	7.40 ± 0.65	1.70 ± 0.13	1.62 ± 0.13
Upper loin (1st–5th)	15.74 ± 1.84	16.62 ± 0.88	3.79 ± 0.39	3.65 ± 0.39
Middle loin (6th–9th)	10.75 ± 1.36	11.96 ± 1.70	2.58 ± 0.16	2.60 ± 0.19
Lower loin (10th–13th)	7.54 ^b^ ± 0.65	8.52 ^a^ ± 0.51	1.82 ± 0.23	1.89 ± 0.32
Chuck flap	4.12 ^b^ ± 0.57	4.89 ^a^ ± 0.46	1.00 ± 0.17	1.08 ± 0.16
Striploin	9.57 ^b^ ± 0.62	11.11 ^a^ ± 1.39	2.31 ± 0.22	2.43 ± 0.32
Chuck roll	20.24 ± 1.67	19.08 ± 3.13	4.87 ^a^ ± 0.29	4.14 ^b^ ± 0.19
Chuck tender	3.37 ± 0.29	3.53 ± 0.46	0.81 ± 0.10	0.77 ± 0.05
Oyster blade	4.40 ± 0.41	5.01 ± 0.65	1.06 ± 0.07	1.09 ± 0.09
Bolar blade	13.89 ± 1.00	14.33 ± 1.29	3.36 ± 0.35	3.14 ± 0.23
Rib blade	1.97 ± 0.23	1.93 ± 0.42	0.48 ± 0.05	0.42 ± 0.09
Upper oyster blade	2.68 ± 0.28	2.64 ± 0.27	0.65 ± 0.10	0.58 ± 0.03
Top round	19.94 ± 1.52	19.74 ± 1.48	4.82 ± 0.56	4.33 ± 0.38
Eye of round	4.56 ± 0.37	4.61 ± 0.61	1.10 ^a^ ± 0.06	1.00 ^b^ ± 0.07
Rump	11.09 ± 0.73	11.71 ± 0.83	2.67 ± 0.22	2.56 ± 0.18
Outside round	13.93 ± 1.06	14.55 ± 1.28	3.36 ± 0.36	3.19 ± 0.25
Outside round head	4.68 ± 0.46	4.20 ± 0.42	1.13 ^a^ ± 0.16	0.92 ^b^ ± 0.09
Knuckle	11.14 ^b^ ± 1.18	12.83 ^a^ ± 1.15	2.69 ± 0.29	2.81 ± 0.29
Tri-tip	2.71 ± 0.15	2.95 ± 0.24	0.65 ± 0.05	0.65 ± 0.09
Brisket point end	22.41 ± 1.92	24.81 ± 4.59	5.39 ± 0.28	5.36 ± 0.35
Brisket point end-deckle off	5.36 ± 1.18	6.30 ± 1.13	1.28 ± 0.22	1.36 ± 0.09
Short blade	8.56 ± 0.97	8.02 ± 1.41	2.06 ^a^ ± 0.22	1.74 ^b^ ± 0.18
Inside skirt	1.64 ^a^ ± 0.18	1.21 ^b^ ± 0.07	0.40 ^a^ ± 0.08	0.27 ^b^ ± 0.03
Thin flank	11.94 ± 1.87	14.59 ± 2.30	2.85 ± 0.17	3.17 ± 0.31
Internal flank plate	3.75 ± 0.48	3.98 ± 0.62	0.90 ± 0.08	0.86 ± 0.05
Flank steak	2.80 ± 0.82	3.56 ± 0.67	0.66 ± 0.13	0.78 ± 0.12
Fore shank	8.44 ± 0.81	8.62 ± 1.09	2.04 ± 0.28	1.88 ± 0.12
Hind shank	8.88 ± 0.84	9.60 ± 0.99	2.14 ± 0.23	2.10 ± 0.12
Heel meat	4.35 ± 0.38	4.39 ± 0.41	1.05 ± 0.12	0.96 ± 0.09
Heel meat center	0.75 ± 0.06	0.82 ± 0.12	0.18 ± 0.02	0.18 ± 0.02
Conical meat	1.35 ± 0.16	1.46 ± 0.21	0.33 ± 0.06	0.32 ± 0.02
Chuck short rib(1st–5th)	6.94 ± 0.61	7.69 ± 0.99	1.67 ± 0.11	1.67 ± 0.07
Short rib (6th–8th)	11.60 ± 0.60	12.74 ± 1.60	2.80 ± 0.20	2.77 ± 0.03
Short rib (9th–13th)	8.97 ± 0.81	9.97 ± 1.35	2.16 ± 0.21	2.17 ± 0.14
Rib finger	18.96 ± 1.51	20.89 ± 2.51	4.56 ± 0.19	4.55 ± 0.14
Hanging tender	1.04 ± 0.11	1.16 ± 0.15	0.25 ± 0.03	0.25 ± 0.02
Outside skirt	1.56 ± 0.19	1.75 ± 0.27	0.37 ± 0.02	0.38 ± 0.03
Neck chain	0.91 ± 0.05	0.92 ± 0.13	0.22 ± 0.03	0.20 ± 0.02
Total meat yield	237.50 ± 19.54	247.44 ± 26.22	57.31 ± 5.72	53.99 ± 2.45

Means within a row with different superscripts (a, b) differ significantly (*p* < 0.05).

**Table 4:** The effect of cattle breed on the chemical composition of the *longissimus lumborum* and *semimembranosus* muscles.

Composition	*M. Longissimus Lumborum*		*M. Semimembranosus*	
	Chikso	Hanwoo	Chikso	Hanwoo
Protein (%)	17.19 ± 1.03	17.18 ± 0.52	17.21 ^b^ ± 0.49	20.43 ^a^ ± 0.10
Moisture (%)	67.74 ^a^ ± 1.29	56.61 ^b^ ± 1.83	70.02 ^a^ ± 0.87	67.49 ^b^ ± 0.45
Intramuscular fat (%)	8.83 ^b^ ± 1.92	23.96 ^a^ ± 2.58	5.44 ^b^ ± 0.92	8.34 ^a^ ± 0.89
Collagen (%)	1.25 ^b^ ± 0.20	2.21 ^a^ ± 0.13	1.25 ^b^ ± 0.14	1.78 ^a^ ± 0.15
Ash (%)	3.42 ^a^ ± 0.27	1.97 ^b^ ± 0.07	3.58 ^a^ ± 0.20	2.35 ^b^ ± 0.11

Means within a row with different superscripts (a, b) differ significantly (*p* < 0.05).

**Table 5:** The effect of cattle breed on the relative percentage of fatty acids in *longissimus lumborum* and *semimembranosus* muscles.

Fatty Acids	*M. Longissimus Lumborum*		*M. Semimembranosus*	
	Chikso	Hanwoo	Chikso	Hanwoo
Myristic acid (C14:0) (%)	3.43 ± 0.28	4.30 ± 0.28	2.79 ^b^ ± 0.20	3.55 ^a^ ± 0.23
Palmitic acid (C16:0) (%)	28.18 ^b^ ± 0.52	31.18 ^a^ ± 0.56	27.22 ^b^ ± 0.52	32.51 ^a^ ± 0.51
Palmitoleic acid (C16:1n7) (%)	4.13 ± 0.49	4.96 ± 0.17	4.07 ± 0.51	3.49 ± 0.36
Stearic acid (C18:0) (%)	11.51 ± 0.61	10.87 ± 0.43	10.71 ± 0.68	11.08 ± 1.02
Cis-Vaccenic acid (C18:1n7) (%)	0.46 ^a^ ± 0.07	0.30 ^b^ ± 0.03	0.49 ^a^ ± 0.08	0.27 ^b^ ± 0.04
Oleic acid (C18:1n9) (%)	49.33 ± 0.80	46.65 ± 0.89	50.51 ^a^ ± 1.00	46.90 ^b^ ± 0.49
Linoleic acid (C18:2n6) (%)	2.37 ^a^ ± 0.38	1.37 ^b^ ± 0.14	3.06 ± 0.74	1.75 ± 0.17
Linolenic acid (C18:3n3) (%)	0.09 ± 0.01	0.06 ± 0.01	0.10 ^a^ ± 0.01	0.06 ^b^ ± 0.01
Gamma linoleic acid (C18:3n6) (%)	0.02 ^a^ ± 0.00	0.001 ^b^ ± 0.00	0.02 ^a^ ± 0.00	0.01 ^b^ ± 0.00
Eicosenoic acid (C20:1n9) (%)	0.16 ± 0.02	0.22 ± 0.02	0.20 ± 0.03	0.20 ± 0.03
Arachidonic acid (C20:4n6) (%)	0.27 ± 0.11	0.05 ± 0.00	0.72 ± 0.29	0.15 ± 0.03
Eicosapetaenoic acid (C20:5n3) (%)	0.00 ± 0.00	0.00 ± 0.00	0.00 ± 0.00	0.00 ± 0.00
Adrenic acid (C22:4n6) (%)	0.05 ± 0.01	0.04 ± 0.01	0.11 ± 0.03	0.05 ± 0.01
Docosahexanoic acid (C22:6n3) (%)	0.00 ± 0.00	0.00 ± 0.00	0.00 ± 0.00	0.00 ± 0.00
SFA (%)	43.12 ^b^ ± 0.70	46.35 ^a^ ± 0.89	40.73 ^b^ ± 0.69	47.13 ^a^ ± 0.60
UFA (%)	56.88 ^A^ ± 0.70	53.66 ^B^ ± 0.89	59.27 ^A^ ± 0.69	52.87 ^B^ ± 0.60
MUFA (%)	54.08 ± 0.92	52.14 ± 0.97	55.27 ^a^ ± 1.34	50.85 ^b^ ± 0.62
PUFA (%)	2.80 ^a^ ± 0.51	1.52 ^b^ ± 0.15	4.01 ± 1.06 ^a^	2.02 ± 0.20 ^b^
n3(%)	0.09 ± 0.01	0.06 ± 0.01	0.10 ^a^ ± 0.01	0.06 ^b^ ± 0.01
n6 (%)	2.72 ^a^ ± 0.50	1.46 ^b^ ± 0.14	3.91 ± 1.05	1.96 ± 0.19
n6/n3	32.40 ± 4.47	25.87 ± 1.87	37.58 ± 8.03	32.25 ± 2.14
MUFA/SFA	1.26 ± 0.04	1.13 ± 0.04	1.36 ^A^ ± 0.05	1.08 ^B^ ± 0.03
PUFA/SFA	0.07 ^a^ ± 0.01	0.03 ^b^ ± 0.00	0.10 ± 0.03	0.04 ± 0.00

SFA: saturated fatty acid; UFA: unsaturated fatty acid, MUFA: monounsaturated fatty acid, PUFA: polyunsaturated fatty acid. Means within a row with different superscripts (a, b) differ significantly (*p* < 0.05).

## Data Availability

Not Applicable.
